# Extracorporeal membrane oxygenation treatment for severe asthma had unexpected adverse effects: a case report

**DOI:** 10.3389/fmed.2023.1294421

**Published:** 2023-11-28

**Authors:** Yun Wang, Weimin Zhang, Xingxing Chen, Xuping Cheng, Xuandong Jiang

**Affiliations:** Intensive Care Unit, Dongyang Hospital of Wenzhou Medical University, Jinhua, Zhejiang,China

**Keywords:** asthma, extracorporeal membrane oxygenation, hemorrhage, hypokalemia, respiratory acidosis

## Abstract

Asthma, a chronic respiratory ailment, affects millions worldwide. Extracorporeal membrane oxygenation (ECMO) has gained traction as a life-saving intervention for patients with severe asthma who are unresponsive to conventional treatments. However, complications associated with ECMO, including electrolyte imbalances and hemorrhage, can have significant clinical implications. This case report highlights a 49 years-old male patient with severe asthma who developed pronounced hypokalemia and hemorrhage following venovenous ECMO (VVECMO) therapy. Despite potassium supplementation, serum potassium levels continued declining before normalizing after 24 h. The patient subsequently experienced gastrointestinal bleeding, cerebral hemorrhage, and extensive cerebral infarction, ultimately resulting in a deep coma. Hypokalemia during ECMO therapy can result from a rapid reduction of carbon dioxide, β-receptor agonist use, corticosteroid use, and diuretic administration. Hemorrhage is another common ECMO complication, often linked to heparin anticoagulation therapy. Clinicians should be aware of potential complications and adopt appropriate prevention and management strategies when using ECMO in patients with severe asthma.

## 1 Introduction

Asthma, a widespread chronic respiratory disease, affects hundreds of millions worldwide (1, 2). It is characterized by airway inflammation, bronchoconstriction, and increased mucus secretion, resulting in episodic symptoms such as wheezing, coughing, chest tightness, and dyspnea. Extracorporeal membrane oxygenation (ECMO) is a life-saving intervention for patients with severe asthma who are unresponsive to conventional treatments (3). This extracorporeal life support temporarily sustains cardiopulmonary function by externally removing carbon dioxide and oxygenating blood (4). In contemporary times, ECMO has gained significant attention as a viable therapeutic alternative for severe respiratory failure arising from diverse etiologies, including acute respiratory distress syndrome, pneumonia, and exacerbated asthma (3, 5, 6). With the widespread adoption of ECMO technology in intensive care units, its application in managing patients with severe asthma has also increased. Several cases have been reported where ECMO has been successfully used to manage severe asthma exacerbations that were refractory to traditional treatments. These cases highlight the potential of ECMO as a therapeutic option in critical asthma management, especially when other interventions have failed (7–9). Despite these potential advantages, ECMO is associated with several complications, including hemorrhage, infection, thrombosis, and electrolyte imbalance (10). Electrolyte imbalance is common in ECMO and is often treated with continuous renal replacement therapy. However, during the literature search, we did not find any report on ECMO-related severe hypokalemia in patients with asthma, which is infrequently documented but can have significant clinical implications.

This case report presents a patient with severe asthma who, following venovenous extracorporeal membrane oxygenation (VVECMO) therapy, manifested a rare and grave complication: pronounced hypokalemia and hemorrhage. This occurrence emphasizes the potential risks of ECMO treatment for patients with severe asthma. It underscores the criticality of closely monitoring and managing electrolyte imbalances and coagulation function in critically ill patients.

## 2 Case description

**Figure 1:**
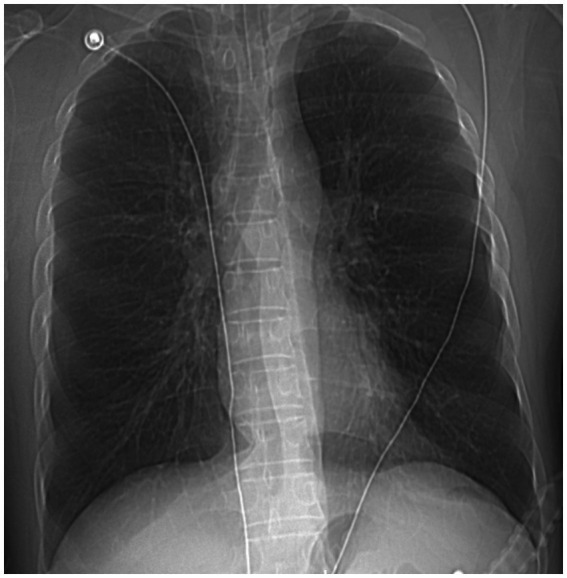
A chest radiograph shows no obvious abnormalities in both lungs.

A 49 years-old man was admitted to our hospital with recurrent chest tightness and dyspnea for over 20 years, exacerbated for 16 h. The patient had a history of asthma for >20 years, intermittent use of traditional Chinese medicine to alleviate the disease (details unknown), which had not been adequately treated, and a history of smoking and drinking for over two decades. Upon emergency admission, the patient presented with a body temperature of 37.5°C, heart rate of 156 beats per minute, respiratory rate of 30 breaths per minute, blood pressure of 185/100 mmHg, and peripheral oxygen saturation of 56%. Other observations included scattered wheezing sounds in both lungs, a regular rhythm, no pathological murmurs detected upon auscultation of each valve, no abdominal tenderness, and no limb edema. Arterial blood gas analysis revealed respiratory acidosis [pH, 7.20; partial pressure of carbon dioxide (pCO_2_), 74 mmHg; partial pressure of oxygen (pO_2_), 54.6 mmHg; bicarbonate (HCO_3_) level, 29.3 mmol/L; and lactate level, 3.8 mmol/L]. The nucleic acid test for severe acute respiratory syndrome coronavirus yielded negative results. Chest radiography revealed no abnormalities in either lung (Figure 1).

The patient was diagnosed with asthma and subsequently treated with nebulized budesonide 1 mg, isopropyl bromide 0.5 mg twice daily, ceftriaxone sodium 2 g intravenous drip once daily for anti-infective therapy, methacrylate sodium succinate 40 mg every 6 h, epinephrine 0.2 mg/h via a micropump, and atracurium 20 mg/h via a micropump injection in conjunction, all of which were ineffective. The patient required immediate intubation and mechanical ventilation and was subsequently transferred to the intensive care unit for further monitoring and treatment. Following mechanical ventilation using the pressure control ventilation mode (frequency: 14 breaths/min, control pressure: 28 cm H_2_O, inspiratory time: 1 s, positive end-expiratory pressure: 10 cm H_2_O, and fraction of inspired oxygen: 100%), and in combination with a comprehensive 3 days regimen of sedation/analgesia, the patient exhibited a progressive decrease in tidal volume to approximately 200 mL, with peak airway pressure reaching 38 mmHg and persistent acidosis. Subsequent blood gas analysis revealed a pH of 6.86, a pCO_2_ of 218 mmHg, a pO_2_ of 194 mmHg, and an HCO_3_ level of 6.9 mmol/L.

After extensive consultations with family members, the patient underwent VVECMO. Additionally, intravenous heparin was administered at 250 U/h for anticoagulation, and the activated partial thromboplastin time was monitored every 4 h to maintain a range of 60–80 s. At the 1 h mark following ECMO, a significant improvement in acidosis was observed, as evidenced by a blood gas pH of 7.36 and PaCO_2_ of 54.8 mmHg. However, hypokalemia was detected at 2.6 mmol/L, prompting the administration of large amounts of rapid intravenous potassium supplementation. Despite this intervention, the serum potassium levels continued to decline, reaching a nadir of 1.7 mmol/L within 7 h before ultimately normalizing after 24 h.

On hospitalization day 4, the patient experienced gastrointestinal bleeding, followed by cerebral hemorrhage and extensive cerebral infarction. After consultation, the family opted for conservative treatment. However, 9 days after admission, the patient’s consciousness remained unimproved, and he was in a deep coma. The family consequently decided to terminate further treatment, and the patient succumbed shortly following his release from medical care.

## 3 Discussion

This case report highlights the potential complications that patients with severe asthma may experience during ECMO therapy, particularly the pronounced hypokalemia and hemorrhage that occurred after VVECMO treatment. Hypokalemia can result in severe consequences, such as life-threatening arrhythmias, muscle weakness, and paralysis, emphasizing the importance of meticulously monitoring and managing electrolyte imbalances in critically ill patients (11).

The principal criterion for implementing ECMO in individuals with asthma is refractory hypercapnic respiratory failure, distinguished by persistently elevated carbon dioxide levels in the bloodstream despite intensive mechanical ventilation and other ancillary interventions (12). Hypokalemia is associated with a rapid reduction in carbon dioxide levels, leading to an expedited exchange of potassium ions between the intracellular and extracellular environments. In particular, the augmented rate of potassium and hydrogen ion exchange causes the translocation of potassium ions into cells as hydrogen ions migrate outward, thereby leading to secondary hypokalemia (13). Hence, it is recommended that the ECMO gas flow be slowly adjusted in patients with persistent hypercapnia if the condition permits, facilitating a gradual reduction in carbon dioxide levels to facilitate the body’s adaptation to alterations in acidity, while closely monitoring electrolyte levels and clinical status (14).

Furthermore, hypokalemia in patients with asthma may be attributed to various factors (Table 1) (15). In particular, patients with asthma frequently use β-receptor agonists such as albuterol that can trigger intracellular potassium ion shifts and consequent hypokalemia (16). The use of corticosteroids, crucial medications in asthma management, may cause hypokalemia through augmented urinary potassium excretion (17). Additionally, diuretic administration during ECMO may contribute to the development of hypokalemia (18). Hemorrhage is a prevalent complication of ECMO; 40% of patients on VVECMO had one or more bleeding events. Hemorrhage is frequently associated with heparin anticoagulation therapy (19, 20). In patients with severe asthma undergoing ECMO therapy, coagulation management should be an essential aspect of the comprehensive treatment plan (21, 22). This case highlights the importance of judicious patient selection, vigilant monitoring, and effective management of complications during ECMO therapy.

**Table 1:** Pharmacologic factors for hypokalemia in patients with asthma.

Medications	Possible corrections
β-receptor agonists (e.g., albuterol)	Monitoring blood potassium levels regularly and supplementation as necessary
Corticosteroids	Evaluation of the necessity of corticosteroid dose and adjustment as needed, frequent monitoring and supplementation of potassium
Diuretics	Reduction of the diuretic dose and supplementation of potassium, administration of potassium-sparing diuretics

In conclusion, ECMO therapy represents a critical intervention for patients experiencing severe asthma and refractory respiratory failure. However, the rapid reduction of carbon dioxide levels after ECMO treatment may lead to severe hypokalemia. In such cases, if the patient’s condition allows, it is advisable to regulate the carbon dioxide reduction rate. As the use of ECMO in patients with asthma becomes increasingly prevalent, clinicians should be cognizant of potential complications, including hypokalemia and hemorrhage, and adopt appropriate measures for prevention and management.

## Data availability statement

The original contributions presented in the study are included in the article/supplementary material, further inquiries can be directed to the corresponding author.

## Ethics statement

The studies involving humans were approved by the Ethics Committee of Dongyang People’s Hospital. The studies were conducted in accordance with the local legislation and institutional requirements. The participants provided their written informed consent to participate in this study. Written informed consent was obtained from the individual(s) for the publication of any potentially identifiable images or data included in this article.

## Author contributions

YW: Writing – original draft. WZ: Conceptualization, Writing – review & editing. XiC: Data curation, Writing – review & editing. XuC: Conceptualization, Writing – review & editing. XJ: Writing – review & editing.
